# A study of Pap smear abnormalities in asymptomatic women attending a tertiary care hospital

**DOI:** 10.6026/973206300221057

**Published:** 2026-02-28

**Authors:** Neelam Singh Raghuwanshi, Debasmita Jana, Akansha Agrawal, Parul Nema, Vishnu Kumar Gupta

**Affiliations:** 1 Department of Obstetrics & Gynaecology, SRVS Medical College, Shivpuri, India; 2 Department of Pathology, SRVS Medical College, Shivpuri, India; 3 Department of Community Medicine, SRVS Medical College, Shivpuri, India

**Keywords:** Pap smear, cytology, asymptomatic screening, cervical intraepithelial lesions, India

## Abstract

Cervical cytological abnormalities detected during asymptomatic screening provide an opportunity for early intervention in premalignant disease. This cross-sectional study enrolled 400 asymptomatic women aged 21-65 years undergoing routine Pap smear screening at a tertiary care hospital. Adequate cytology was obtained in 97 % of participants, with 87.5% showing NILM, 5% inflammatory changes and 4.5% epithelial cell abnormalities (ASC-US 2.5%, LSIL 1.25%, HSIL 0.75%). Abnormalities were more frequent in women aged ≥ 40 years, multiparous women and those of lower socioeconomic status (p < 0.05). Targeted screening and structured follow-up are essential to improve early detection and prevention of cervical neoplasia.

## Background:

Cervical cancer continues to be a significant public health concern, particularly in low- and middle-income countries, where it ranks among the foremost causes of cancer-related morbidity and mortality in women [1]. Although well- established preventive measures such as HPV vaccination and cervical cytology screening exist, their implementation remains suboptimal in many developing regions [1]. The Papanicolaou (Pap) smear test serves as a fundamental tool in the early detection of cervical intraepithelial neoplasia, enabling timely intervention before progression to invasive malignancy [2]. Routine screening using Pap smears has demonstrated effectiveness in identifying a range of cytological abnormalities, often in asymptomatic individuals, thereby reducing the burden of advanced-stage cervical cancer [2]. In the Indian context, studies conducted at tertiary healthcare institutions have reported cytological abnormalities in 4% to 8% of asymptomatic women undergoing routine screening [3, 4]. Among these, inflammatory smears are most frequently observed, followed by epithelial cell abnormalities such as atypical squamous cells of undetermined significance (ASC-US), low-grade squamous intraepithelial lesions (LSIL) and high-grade squamous intraepithelial lesions (HSIL), which collectively account for approximately 1% to 6% of all cases [3, 4]. Comparable findings have been documented in Middle Eastern populations, where epithelial abnormalities are identified in 3% to 6% of screened women [5].

Multiple socio-demographic and reproductive factors have been implicated in increasing the risk of cervical cytological abnormalities. These include limited educational attainment, early age at marriage and first childbirth, high parity, multiple sexual partners, poor genital hygiene and persistent infections such as bacterial cervicitis [6]. Despite the well- recognized benefits of early detection, awareness regarding cervical cancer screening remains limited and the uptake of Pap smear testing is notably low among eligible women in India, particularly in rural and underserved areas [7]. Therefore, it is of interest to describe the prevalence, pattern and associated socio-demographic factors of Pap smear abnormalities among asymptomatic women attending a tertiary care hospital.

## Materials and Methods:

This descriptive, cross-sectional study was carried out at a tertiary care teaching hospital. The study population comprised 400 asymptomatic women aged between 21 and 65 years, who presented for routine gynecological check-ups or participated in cervical cancer screening camps organized by the hospital. Women presenting with active vaginal symptoms (such as discharge or bleeding), those with visible cervical lesions on per speculum examination, a known history of cervical intraepithelial neoplasia, carcinoma cervix, or those previously treated for cervical pathology were excluded to minimize confounding factors. Upon obtaining informed written consent, each participant was interviewed using a structured questionnaire to collect demographic information (including age, education and socio- economic status) as well as obstetric and gynecologic history (such as parity, age at first childbirth and contraceptive use). Cervical cytological samples were collected using Ayre's spatula for the ectocervix and an endocervical brush for the endocervical canal. The collected smears were immediately fixed in 95% ethanol and subsequently stained using the standard Papanicolaou staining technique. All cytological slides were interpreted and reported in accordance with the 2014 Bethesda System [8], which classifies findings into the following categories: Negative for Intraepithelial Lesion or Malignancy (NILM), Atypical Squamous Cells of Undetermined Significance (ASC-US), Low-grade Squamous Intraepithelial Lesion (LSIL), High-grade Squamous Intraepithelial Lesion (HSIL), Atypical Glandular Cells (AGC) and Squamous Cell Carcinoma (SCC). Smears were labeled as unsatisfactory for evaluation if they were poorly fixed, inadequately cellular, or significantly obscured by blood or inflammation. Statistical analyses were conducted using IBM SPSS software version 25.0. Descriptive statistics including frequencies and percentages were used to summarize categorical variables. The association between abnormal cervical cytology and various socio-demographic and reproductive variables-such as age group, parity and socio-economic status-was assessed using the Chi-square test. A p-value of less than 0.05 was considered statistically significant.

## Results:

The demographic profiles of patients are presented in Table 1. Among 400 screened women, the unsatisfactory rate was low (3.0%) and most samples were adequate for cytological interpretation (Table 2). The majority of smears were NILM, with inflammatory changes accounting for 5% and epithelial abnormalities detected in 4.5% (ASC-US, LSIL, HSIL combined) (Table 2). There were no cases of AGC or invasive carcinoma. Risk factor analysis (Table 3) revealed that increased age (>40 years), multiparity and low socioeconomic status were significantly associated with detection of epithelial abnormalities. These findings confirm that certain demographic factors correlate with cytological risk even in asymptomatic populations.

## Discussion:

In concordance with existing regional Indian data, the present study observed a prevalence of epithelial cell abnormalities at 4.5% among asymptomatic women undergoing routine screening. This figure aligns well with previously reported prevalence rates ranging from 3% to 6% in studies conducted at tertiary care centers across the country [9, 10]. Furthermore, the observed low rate of unsatisfactory smears indicates the adequacy of sample collection techniques and adherence to standard cytological protocols, ensuring diagnostic reliability. Age-wise analysis revealed that women over 40 years of age exhibited a significantly higher frequency of epithelial abnormalities. This finding supports the age-related trend reported in population-based screening studies, where the incidence of squamous intraepithelial lesions (SIL) tends to rise with advancing age due to cumulative exposure to risk factors and potential decline in immune surveillance [11, 12]. Additionally, the study identified a statistically significant association between cytological abnormalities and multiparity as well as low socio-economic status. These associations are consistent with findings from community-based surveys in South India, which have reported that high parity and limited educational attainment significantly increase the risk of cervical dysplasia, likely due to repeated cervical trauma, hormonal influences and poor access to reproductive health services [13].

The majority of detected abnormalities in this cohort were low-grade lesions, such as ASC-US and LSIL. These lesions are often transient and have a high likelihood of spontaneous regression, particularly in younger women. Nonetheless, their identification is clinically relevant, as they warrant close monitoring through repeat cytology or adjunctive HPV DNA testing to ensure early intervention if progression occurs. Importantly, no cases of HSIL or invasive carcinoma were documented in this sample, a finding consistent with large-scale hospital-based audits in India that have reported HSIL prevalence rates between 0.5% and 0.8% among asymptomatic women undergoing routine screening [14,15]. The findings of this study highlight the clinical value of routine Pap smear screening, even among women without symptoms, by facilitating the early detection of premalignant changes. This is particularly pertinent in resource-constrained settings, where lack of awareness and limited access to preventive services have contributed to delayed diagnosis and poor outcomes. Prior studies have underscored the widespread deficiency in awareness and screening uptake, further emphasizing the need for enhanced community education and integration of opportunistic screening into routine gynecological care [7]. Strengths of this study include a methodologically robust sample size, the deliberate exclusion of symptomatic individuals to specifically assess screening effectiveness in an asymptomatic cohort and the use of the Bethesda 2014 system, ensuring standardization and reproducibility of cytological interpretation. However, certain limitations should be acknowledged, including the absence of high-risk HPV DNA testing, which could have provided etiological confirmation of lesions; lack of colposcopic evaluation for cytologically abnormal cases; and the unavailability of follow-up data to ascertain the natural course or resolution of low-grade lesions. Finally, supporting the notion of relatively low prevalence of cytological abnormalities in asymptomatic screening populations, Jadav *et al.* reported in a tertiary care cohort from Ahmedabad that among 487 Pap smears screened per the Bethesda 2014 system, epithelial abnormalities (including ASC-US, LSIL and HSIL) were rare, with only one case each of ASC-US, LSIL and HSIL identified alongside two invasive squamous cell carcinomas [Jadav *et al.* A study of cervical pap smears in a tertiary care hospital of Ahmedabad, Gujarat, India (2019)]. This finding further underscores the variability in detection rates across Indian screening settings while affirming the utility of Pap smear screening in early lesion identification [16].

## Conclusion:

We show that Pap smear screening can effectively detect early cytological abnormalities even in asymptomatic women. A significant proportion of these abnormalities were low-grade lesions, indicating a potential window for preventive intervention. Age over 40 years, multiparity and lower socioeconomic status was found to be associated with increased risk. Routine cervical cancer screening programs targeting at-risk populations are essential to reduce the burden of invasive cervical disease. Strengthening awareness and access to Pap smear services can play a crucial role in early detection and timely management.

## Figures and Tables

**Table 1:** Demographic profile among study subjects (N=400)

**Variable**	**Category**	**N (%**
Age (years)	21-30	120 (30.0)
	31-40	150 (37.5)
	41-50	90 (22.5)
	51-65	40 (10.0)
Parity	Nulliparous	180 (45.0)
	Multiparous	220 (55.0)
Socioeconomic	Low	160 (40.0)
status	Middle/High	240 (60.0)

**Table 2:** Pap smear findings among study participants

**Cytology Category**	**N (%)**
Unsatisfactory samples	12 (3.0)
NILM	350 (87.5)
Inflammatory changes only	20 (5.0)
ASC-US	10 (2.5)
LSIL	5 (1.25)
HSIL	3 (0.75)
AGC	0
SCC	0

**Table 3:** Association with risk factors

**Factor**	**Abnormal Cytology (n=18)**	**NILM (n=350)**	**p-value**
Age >40 years	12	118	0.021*
Multiparity (≥2)	14	206	0.034*
Low socioeconomic	10	150	0.045*
*Statistically significant
